# Molecular mobility on graphene nanoroads

**DOI:** 10.1038/srep12848

**Published:** 2015-08-05

**Authors:** Mehdi Jafary-Zadeh, Yong-Wei Zhang

**Affiliations:** 1 Institute of High Performance Computing, A*STAR, Singapore 138632.

## Abstract

We study molecular mobility on a graphene nanoroad (GNRD), a pristine graphene strip embedded in between two hydrogenated graphene domains serving as a nanoscale pathway for transporting admolecules. Our molecular dynamics simulations using a prototype physisorbed C_60_ admolecule demonstrate that the proposed GNRD is able to confine the diffusive motion of the admolecule within the nanoroad up to a certain temperature, depending on its width and edge type. Within the confinement regime, the width and edge-type of the GNRD also play an important role in the molecular motion. Specifically, when the GNRD width is narrower than the admolecule diameter, the admolecule performs one-dimensional hopping motion along the nanoroad. When the GNRD width is larger than the admolecule diameter, the admolecule moves only along one of its edges at low temperatures, and shuffle between two edges at high temperatures. We further show the admolecule motion on the zigzag-edged GRND is faster than that on the armchair-edged GRND with the same width and at the same temperature. These results can be well explained by analysing the potential energy surfaces of the systems. Since such hydrogenated graphene nanostructures have been experimentally realized, our results provide a valuable reference for constructing molecular conveyor circuits.

Precise control of the position and mobility of nanoscale building-blocks, such as atoms, molecules and clusters, is an ultimate challenge in nanotechnology^1^ with ubiquitous applications in nanodevice fabrication^2^, catalysis^3^, information technology^4^ and nanomedicine^5^. To this end, construction of nanoscale pathways whose function is analogous to railway tracks or highways would be essential for precise, efficient, and readily automated delivery of these building-blocks^6^. Moreover, from the fundamental point of view, understanding the mechanisms of molecular motion is also an intriguing task in physics and surface science.

Graphene is electrically and thermally conductive, chemically inert, and mechanically robust. Besides, the kinetic friction of physisorbed molecules on graphene is significantly low, which is desirable for efficient and high speed molecular transportation^7^,^8^,^9^. Hence, graphene-based materials are promising for constructing mass conveyer systems by applying a temperature gradient or external electric field^10^,^11^,^12^,^13^. For example, carbon nanotubes (CNTs) have received special attention to transport nanofluids^10^,^14^. Furthermore, it has been demonstrated that graphene nanoribbons (GNRs) can be employed to transport the adsorbed molecules^15^,^16^,^17^,^18^. The potential energy barrier at the edges of a GNR effectively confines the molecular motion along the nanoribbon^18^. As an advantage to CNTs, the shape of GNRs can be engineered by the lithography approaches^19^. However, cutting and integrating the easily deformable “flimsy” GNRs is a challenge to the implementation of sophisticated mass transport circuits^15^. Hence, efforts are needed to circumvent this technical obstacle and introduce alternative highways for molecular transfer.

Chemical functionalization of graphene, especially with hydrogen, has been shown to be an effective approach for manipulating its electronic and magnetic properties^20^,^21^,^22^. Previous studies on the functionalized graphene have been performed mainly on the mechanical and physical properties of the material^23^,^24^. Recently, application of patterned hydrogenation on graphene for molecular packing has also been suggested^25^. Moreover, the effect of hydrogenation of graphene on the molecular mobility of a physisorbed molecule has been studied^26^. It has been shown that random hydrogenation of graphene leads to a drastic reduction of admolecule mobility due to the strong effect of hydrogenation on the potential energy surface of the system^26^. This finding raises an interesting question: Is it possible to utilize the hydrogenation of graphene to construct a “nanoroad” with a pristine graphene strip being embedded in between two hydrogenated domains without the need for cutting and assembling? Remarkably, this question has been recently addressed^27^,^28^. Using first principles calculations, the feasibility of constructing graphene nanoroads (GNRDs) with desired armchair or zigzag edges embedded between two domains of graphane (fully hydrogenated graphene) was illustrated and their electronic and magnetic properties were studied^27^. Moreover, electron beam lithography (EBL) technique was employed to experimentally realize the hybrid superlattices of hydrogenated graphene with desirable geometries^28^.

In the present work, we perform molecular dynamics (MD) simulations to study the diffusive motion of a prototypical physisorbed admolecule (C_60_) on a graphene nanoroad (GNRD). More specifically, we would like to address the following questions: (1) Can the GNRD confine the motion of a C_60_ admolecule? (2) How does the edge type of the GNRD affect the molecular mobility? (3) How does the width of the GNRD affect the molecular mobility? (4) How does the temperature affect the molecular mobility? Answers to these questions would be useful for finding robust approaches to conveying nanoscale building-blocks and controlling their motion.

## Results and Discussion

**Figure 1:**
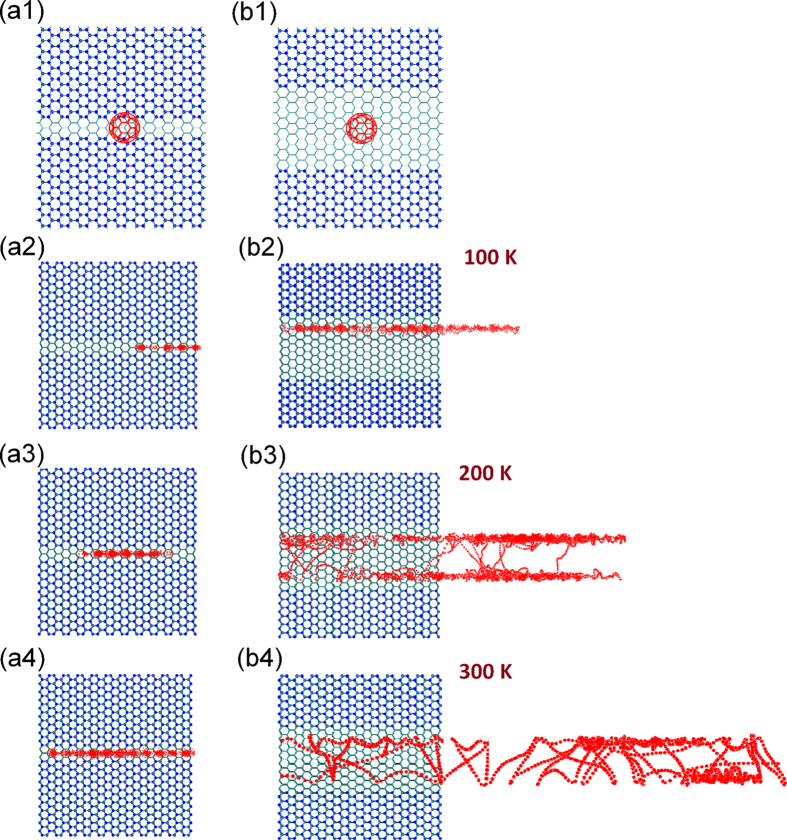
Initial configurations of the C_60_ admolecule on the armchair-edged GNRDs with width of 5 Å (**a1**), and 20 Å (**b1**). The trajectories of the C_60_ admolecule centre of mass (COM) on these two nanoroads at different temperatures of 100 K, 200 K, and 300 K are also presented in (**a2**,**b2**), (**a3**,**b3**), and (**a4**,**b4**), respectively.

First, we examine the trajectories of the C_60_ centre of mass (COM) on the armchair-edged GNRD at different temperatures. Simulation results are shown in Fig. 1, in which the C_60_ COM trajectory is presented in red, the hydrogen atoms are presented with blue circles, and the graphene honeycomb structure is presented in green. The left side panels (Fig. 1(a2–a4, [Fig f3], [Fig f4])) show the C_60_ trajectories on a 5 Å width armchair-edged nanoroad at 100 K, 200 K, and 300 K, respectively. These trajectories illustrate that a 5 Å width nanoroad, which is narrower than the diameter of the C_60_ admolecule (with the mean atom-to-atom diameter of ~7.1 Å)^29^, is able to confine the surface diffusion of the C_60_ along the nanoroad up to the room temperature. The right side panels (Fig. 1(b2–b4)) show the typical trajectories of the C_60_ admolecule on an armchair-edged nanoroad with a width of ~20 Å at 100 K, 200 K and 300 K, respectively. It is seen that the nanoroad, which is wider than the diameter of the admolecule, can also confine the molecular motion up to the room temperature. However, depending on temperature, the confined motion in this case shows two distinct regimes: At low temperatures (see Fig. 1(b2)), the C_60_ admolecule diffuses only along one of the edges. In this regime, the diffusion of the admolecule along the edge is via the hopping mechanism between the adjacent adsorption sites of the edge. This indicates that the edge of the GNRD plays the role as an “adsorbing-wall” in the motion of the admolecule. At high temperatures, the C_60_ admolecule “switches” its motion between the two edges of the GNRD (See Fig. 1(b3,b4)). It can be seen that in this “switching” motion, the duration of sticking intervals is reduced and the admolecule exhibits a quasi-continuous Brownian motion along the nanoroad edges. Our simulations show that the transition temperature between these two regimes for the armchair-edged GNRD occurs at around 150 K. The physics behind the scene of adsorption of the C_60_ admolecule to the edges of a GNRD will be discussed later.

**Figure 2:**
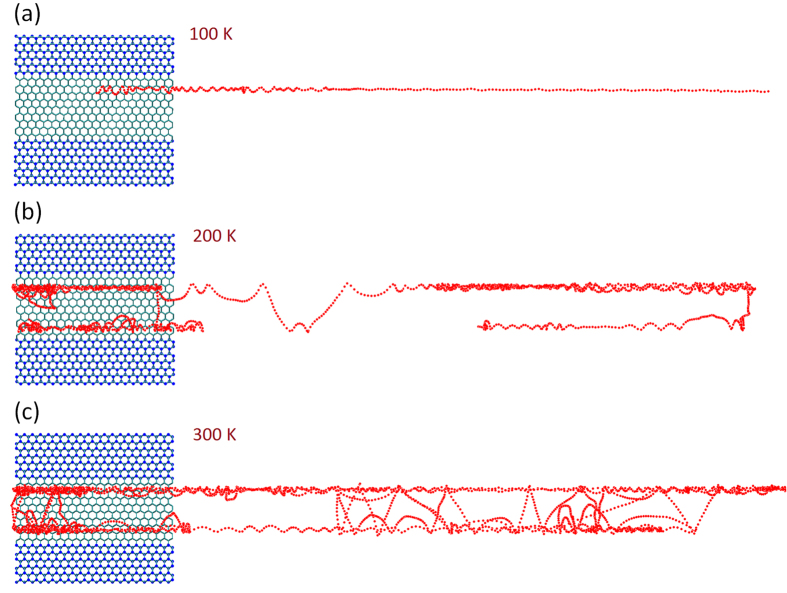
Trajectories of the C_60_ admolecule centre of mass on the zigzag-edged GNRD with a width of about 20 Å at temperatures of (**a**) 100 K, (**b**) 200 K, and (**c**) 300 K.

Next, we examine the trajectories of the C_60_ COM on the zigzag-edged GNRD at different temperatures. Figure 2(a–c) shows the trajectories of the C_60_ admolecule on a zigzag-edged nanoroad with a width of ~20 Å at 100 K, 200 K and 300 K, respectively. It can be seen that, similar to the armchair-edged GNRD as shown in Fig. 1(b2–b4), the zigzag-edged GNRG also confines the molecular mobility up the room temperature. Similar to the GNRDs with armchair-edges, there also exist two distinct regimes depending on temperature, that is, the edge hopping regime at low temperatures (see Fig. 2(a)), and the quasi-continuous Brownian motion at high temperatures (see Fig. 2(b,c)). We found that for the zigzag-edged GNRD, the transition occurs at about 125 K. A comparison between Fig. 1(b2) and Fig. 2(a) reveals an apparent difference in the molecular motion between the armchair and zigzag edges. Figure 1(b1) clearly shows the intervals between hopping events along the armchair-edged GNRD at 100 K; while Fig. 2(a) shows a smoother trajectory of the C_60_ admolecule along the zigzag edge at the same temperature. Hence, the C_60_ admolecule along the zigzag edge has a higher mobility than that along the armchair edge. For comparison, we have provided two Supplementary Movies to show the difference in the diffusion of C_60_ admolecule on these two types of GNRDs at 100 K.

**Figure 3 f3:**
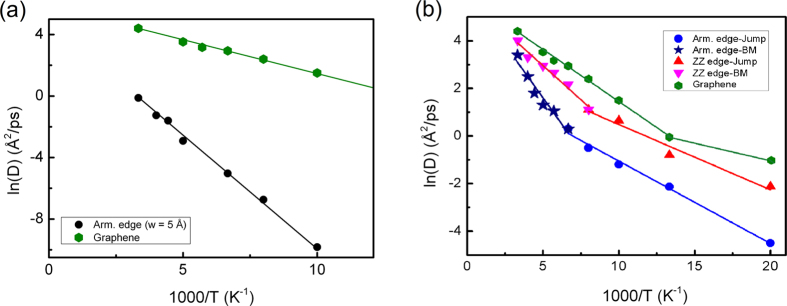
The Arrhenius analysis of the surface diffusion coefficient, *D*, of the C_60_ admolecule on the GNRDs. (**a**) The analysis on the 5 Å armchair-edged GNRD indicates only one regime of surface diffusion (*i.e*. one-dimensional hopping) up to 300 K. For the comparison of the molecular mobility, the values of *D* on the pristine graphene are also plotted here. (**b**) The Arrhenius analysis of *D* on the armchair- and zigzag-edged GNRDs with the widths of ~20 Å indicates that in the temperature range of up to 300 K, these systems show two regimes of diffusion: hopping motion (jumping) along one of the two edges at the lower temperatures, and a Brownian motion along both edges at higher temperatures. The corresponding prefactor and activation energies of these regimes are given in Table 1. The values of *D* on the pristine graphene are also plotted in (**b**) showing a transition at about 75 K between two regimes of Brownian motion^7^,^8^.

To quantify the molecular mobility on the GNRDs, we calculated the diffusion coefficient, *D*, of the C_60_ using the best linear fit to its mean square displacement (MSD) curve. The values of the logarithm of *D* versus the inverse of temperature, *T*, are plotted in Fig. 3. In the case of nanoroads narrower than the C_60_ diameter, Fig. 3(a) shows the results for the armchair-edged GNRD with the width of 5 Å. In this figure, the values of *D* for the C_60_ on the infinite pristine graphene are also plotted for comparison. The widely used Arrhenius relation is employed to analyse the temperature-dependence of *D* as the following:


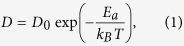


where, *T* is the temperature, *k*_*B*_ is the Boltzmann constant, *D*_*0*_ and *E*_*a*_ are the prefactor and activation energy of the diffusion process, respectively. Figure 3(a) shows that for this case, the values of *D* can be described by a single linear fit with *E*_*a*_ = 0.14 eV and *D*_*0*_ = 128.5 Å^2^/ps, indicating that there is only a single diffusive regime in the whole temperature range up to 300 K. This is in agreement with the trajectories of C_60_ in Fig. 1(a2–a4), which show that up to room temperature, the C_60_ diffusion on the 5 Å armchair-edged GNRD is through a one-dimensional thermally activated jump (hopping) mechanism. Here, it is noted that the narrowest armchair and zigzag GNRDs used in this study had the width of ~5 Å and ~4 Å, respectively. Our analysis of the trajectory of C_60_ admolecule on the 4 Å zigzag GNRD indicates that at temperatures lower than 200 K, the admolecule also performs a one-dimensional jump mechanism. However, the 4 Å zigzag GNRD cannot confine the admolecule at 200 K and above.

In the case of the GNRDs wider than the admolecule diameter, Fig. 3(b) shows the Arrhenius analysis of *D* for the armchair- and zigzag-edged GNRDs with the width of ~20 Å (as shown in Fig. 1(b) and Fig. 2). Figure 3(b) clearly shows that the values of *D* for both armchair- and zigzag-edged GNRDs deviate from a single linear fit in the temperature range of 50 K to 300 K. Indeed, *D* is found to follow two Arrhenius relations for both the zigzag- and armchair-edged GNRDs. The corresponding values of *D*_*0*_ and *E*_*a*_ for these two systems are summarized in Table 1. The change in the Arrhenius relation can be explained by the transition of the surface diffusion regime from the hopping mechanism along a single edge at the lower temperatures to the quasi-continuous Brownian motion along both edges at high temperatures. Here, it is worth noting that according to Fig. 3(b), two Arrhenius relations also exist for the diffusion on pristine graphene, corresponding to two Brownian regimes with different kinetic friction mechanisms in this system^7^,^8^.

**Figure 4 f4:**
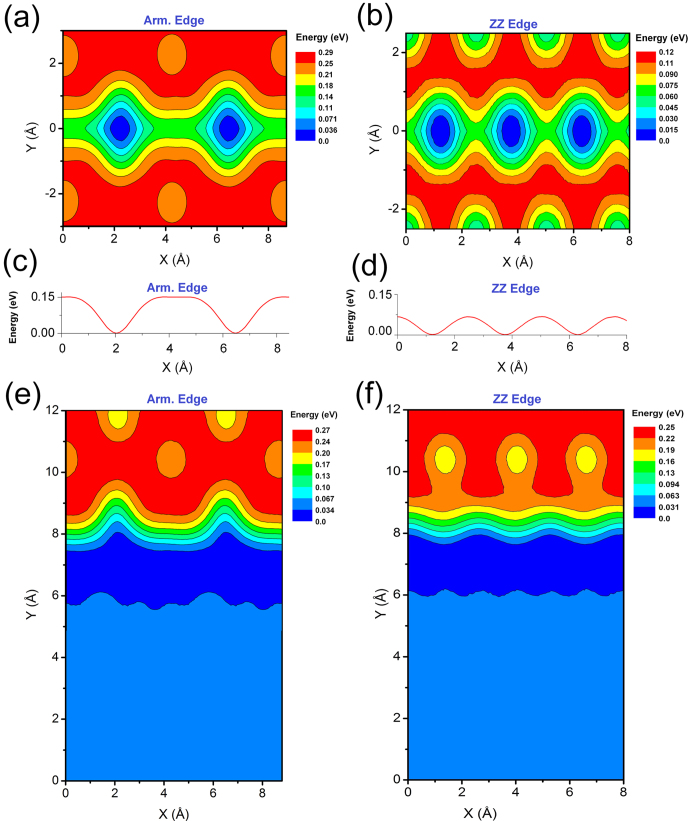
Potential energy surface (PES) of the C_60_ on (**a**) the 5 Å armchair-edged GNRD, and (**b**) the 4 Å zigzag-edged GNRD. (**c**,**d**) The energy profiles along the central line (*y* = 0) of the GNRDs in (**a**,**b**) respectively. (**e**,**f**) The PES of the C_60_ on the GNRD with a width of about 20 Å and different edge types: (**e**) armchair, and (**f**) zigzag. In (**e**,**f**), due to the symmetry of the systems in the *y* direction (perpendicular to the edges), only half of the PES is presented, that is, 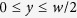.

To understand the physics behind the scene of edge effects on the motion of the C_60_ admolecule on GNRDs, we further investigate the potential energy surface (PES) of the C_60_/GNRD system. Figure 4 shows the PES contour plots of the C_60_ on the armchair- and zigzag-edged GNRDs. In this figure, the horizontal axis (*x* direction) represents the position of the C_60_ COM parallel to the GNRD edge and the axis origin is an arbitrary point along the GNRD. The vertical axis (*y* direction) represents the position of the C_60_ COM perpendicular to the edge of the GNRD and the axis origin is located at the centre of the GNRD with an equal distance from both edges. The colour spectrum of the contour plots represents the level of potential energy of the system with deep-blue for the lowest energy level and red for the highest energy level. Figure 4(a,b) show the PES for the armchair- and zigzag-edged GNRDs with the width of 5 Å and 4 Å, respectively. The corresponding energy profiles along the central line (*y* *=* *0*) of these nanoroads are shown in Fig. 4(c,d). It can be seen that for the GNRDs narrower than the C_60_ diameter, the adsorption sites are along the centre of the nanoroad, providing a one-dimensional pathway for the hopping mechanism between these sites. Figure 4(c) shows that the depth of the adsorption sites along 5 Å armchair nanoroad is about 0.15 eV, which is in good agreement with *E*_*a*_ = 0.14 eV obtained from the Arrhenius analysis of this system as shown in Fig. 3(a) and the one-dimensional hopping trajectory as shown in Fig. 1(a2). Figure 4(c,d) also demonstrate that the adsorption sites along the armchair-edged GNRD are deeper and more separated from each other than those along the zigzag-edged GNRD. This finding explains the slower molecular motion along the armchair-edged nanoroad. Furthermore, a comparison between Fig. 4(a,b) reveals that the height of energy barrier at the edges of the 4 Å zigzag nanoroad is ~0.1 eV, which is smaller than the height of the energy barrier at the edges of 5 Å armchair nanoroad, which is ~0.3 eV (see the energy scales in these figures). Hence, in contrast with the 4 Å zigzag GNRD, the 5 Å armchair nanoroad confines the molecular motion of the C_60_ in the whole temperature range of this study up to the room temperature.

Figure 4(e,f) show the PES for the armchair- and zigzag-edged GNRDs, respectively, where the nanoroad width (~20 Å) is larger than the admolecule diameter. Due to the symmetry of these systems in the *y* direction, only half of the PES, that is, 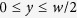, is presented. Figure 4(e,f) show that in both armchair- and zigzag-edged GNRDs, there is a high energy barrier (almost the same height) at the GNRD edge (red and orange), which prevents the C_60_ from escaping from the GNRDs. These figures also show that near the GNRD edge, there is a channel (deep-blue) with the lowest potential energy and the maximal depth is in the order of 30 meV. Consequently, at low temperatures, the C_60_ admolecule tends to stay in this channel and diffuses close to the GNRD edge as can be seen in the trajectories of Fig. 1(b2) and Fig. 2(a). While the C_60_ admolecule diffuses along this low energy channel, it interacts with the potential energy corrugations at the edge of the GNRD, leading to the hopping motion only along one of the GNRD edges at lower temperatures. Clearly, the profile of the PES at the GNRD edges controls the dynamics of the C_60_ diffusion.

A comparison between the PES of the armchair-edged and zigzag-edged GNRDs (Fig. 4(e,f), respectively) indicates that the adsorption sites along the armchair edge are more separated from each other than that along the zigzag edge. As a result, the C_60_ admolecule can hop between the adjacent sites more easily along the zigzag edge than along the armchair edge. This is consistent with the difference in the trajectory of the C_60_ admolecule between the zigzag-edged (see Fig. 2(a)) and armchair-edged GNRD (see Fig. 1(b1)). This also explains the higher diffusion coefficient of the C_60_ admolecule along the zigzag-edged GNRD, especially at lower temperatures where the GNRD edge has more important effect on the molecular mobility (see Fig. 3(b)). By increasing the temperature, the C_60_ admolecule has a higher thermal energy to overcome the energy barrier of the adsorption sites and performs a quasi-continuous Brownian motion along the edge.

**Figure 5:**
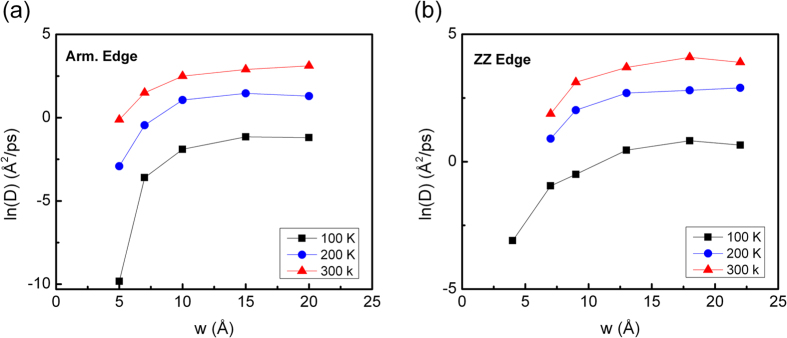
Diffusion coefficient, *D*, of the C_60_ admolecule as a function of the GNRD width, *w*, along (**a**) armchair-, and (**b**) zigzag-edged GNRDs.

We further examine the diffusivity of the C_60_ admolecule on the GNRD as a function of its width, *w*. The variation of diffusion coefficient, *D*, as a function of *w* is plotted in Fig. 5(a,b) at different temperatures for the armchair- and zigzag-edged GNRD, respectively. Here, we recall that in our simulations, the 4 Å zigzag GNRD cannot confine the admolecule at 200 K and above. Hence, there is no data for the *D* values at 200 K and 300 K for this nanoroad. Moreover, Fig. 5 shows that once the width of the GNRD exceeds 10 Å (which is wider than the diameter of the C_60_ admolecule), the value of *D* reaches a plateau. This is due to the fact that the edges of the wide GNRDs control the molecular mobility. Our above results clearly show that the molecular mobility on GNRDs is both edge type- and temperature-dependent.

Production of high-quality and large-area graphene^30^,^31^ together with advanced lithographic technologies have made the fabrication of complex hydrogenated graphene structures possible^28^. In the present work, we showed that such hybrid structures can be used as molecular conveyor highways. Hence, our findings shed light on the possible means to precisely control the molecular mobility. Moreover, the GNRDs studied here are promising for confining and ‘wiring’ the physisorbed molecules into complicated molecular circuits, which is an active research area in nanoelectronics^32^, and other nanoelectromechanical systems (NEMs), such as molecular sensors^33^,^34^, photovoltaic devices^35^, and data storage systems^36^.

## Summary

In summary, we employed molecular dynamics simulations to investigate the feasibility of “nanoroad”, a pristine graphene strip embedded between the two graphane (fully hydrogenated graphene) domains, as a molecular conveyor highway. Our results showed that the motion of a prototypical physisorbed C_60_ admolecule can be confined on the GNRDs. When the GNRD is wider than the diameter of the C_60_ admolecule, the C_60_ only moves along one of the GNRD edges at low temperatures. With increasing the temperature (approximately at 125 K for the zigzag- and 150 K for the armchair-edged GNRD), the C_60_ admolecule is able to shuffle between two edges. Furthermore, when the GNRD width is narrower than the admolecule diameter, the admolecule performs one-dimensional hopping motion along the nanoroad. We further showed that the edge type plays an important role in the molecular mobility, and the admolecule motion on the zigzag-edged nanoroads is faster than on the armchair-edged nanoroads. With increasing the width beyond the diameter of the C_60_ admolecule, the molecular mobility reaches a plateau with the magnitude of the zigzag-edged GNRD being higher than that of the armchair-edged GNRD. Recently, the hybrid graphene superlattices via hydrogenation have been experimentally realized. Hence, our findings here provide a practical guideline for experimentalists to fabricate nanoscale highways.

## Methods and Model

We performed molecular dynamics (MD) simulations using Large-scale Atomic/Molecular Massively Parallel Simulator (LAMMPS)^37^ and the Adaptive Intermolecular Reactive Empirical Bond Order (AIREBO) potential^38^. AIREBO is one of the most successful potentials applied to model both chemical reactions and intermolecular interactions in hydrocarbon systems and graphene based materials^39^,^40^. Here, it should be noted that the adsorption of C_60_ on graphene and graphane surfaces is through the van der Waals interactions. In AIREBO, these interactions are parameterized using the Lennard-Jones (LJ) formulation and a cutoff distance of 10.2 Å. The AIREBO has been successfully used to predict the graphite interlayer separation of 3.354 Å, as well as the adsorption energy of C_60_ on graphene, in agreement with experiments and first-principles calculations (~0.8 eV)^38^,^41^.

Our computational model consists of a single C_60_ admolecule on graphene nanoroads with the variation of width, *w*, up to ~20 Å and also edge type, that is, zigzag or armchair. The simulations were run at the temperature range of 50 K to 300 K (room temperature). GNRDs were constructed via partial hydrogenation of a graphene sheet in a patterned manner so that the graphene road with the desired width was embedded between two graphane (fully hydrogenated graphene) domains as shown in Fig. 1 and explained in Ref. 27. It should be noted that there are two possibilities of graphane according to the configuration of the hydrogen atoms: chair- and boat-like configurations^42^. In this work, we used the chair-like configuration because, energetically, it is more stable^42^.

Periodic boundary conditions (PBCs) were applied along the in-plane directions of the simulation cell. The PBC along the GNRD length (*x* direction) represents an infinitely long nanoroad. The C_60_ admolecule was initially positioned at the centre of each GNRD (equal distance from its edges) and at the equilibrium distance (about 3.1 Å) on top of the substrates in such a way that one of its hexagon faces was oriented parallel to the hexagons of the GNRD. Figure 1(a1,b1) show the initial configurations of the C_60_ admolecule on the armchair-edged GNRDs with widths of 5 Å and 20 Å, respectively. The hydrogen atoms are denoted by blue circles, and the graphene honeycomb structure is denoted in green.

To compare the surface diffusion of C_60_ on GNRDs with that of C_60_ on infinite graphene, a series of simulations were performed for the C_60_/graphene system in the same way as the previous works^7^,^8^,^9^. At the beginning of each simulation, we performed energy minimization using Polak-Ribiere conjugate gradient (CG) method as implemented in LAMMPS package. After energy minimization, the velocities of the atoms in the system were assigned with the desired temperature following the Maxwell-Boltzmann distribution in such a way that no initial aggregated angular and linear momenta were imposed to the system. The microcanonical ensemble was employed for the simulations. The time step of the Verlet algorithm to integrate the equations of motion was 1 fs. Each trajectory calculation was started with a 100 ps thermal equilibration. Then we run the simulations for up to 20 ns to extract the data for further analysis.

The trajectories of the C_60_ COM on the GNRDs were obtained from the MD simulations. At a sufficiently long-time scale, the component of mean square displacement of the C_60_ COM parallel to the GNRD edge, *MSD*, scales linearly with time and the corresponding diffusion coefficient, *D*, can be obtained as a measure for molecular mobility using the best linear fit according to:


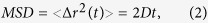


where, *Δr* is the admolecule COM displacement parallel to the GNR edge, *t* is time, and <.> denotes the ensemble or time averaging^43^,^44^,^45^.

## Additional Information

**How to cite this article**: Jafary-Zadeh, M. and Zhang, Y.-W. Molecular mobility on graphene nanoroads. *Sci. Rep*. **5**, 12848; doi: 10.1038/srep12848 (2015).

## Supplementary Material

Supplementary Information

Supplementary Movie S1

Supplementary Movie S2

## Figures and Tables

**Table 1:** The Arrhenius parameters for different diffusive regimes and their corresponding temperature ranges on both armchair- and zigzag-edged GNRDs with a width of about 20 Å.

Nanoroad Edge Type	D_0_ (Å^2^/ps)	E_a_ (eV)	Temperature range (K)
Armchair (Arm.)	11.1	0.03	T < 150
	384.46	0.076	T > 150
Zigzag (ZZ)	25.27	0.02	T < 125
	424.11	0.05	T > 125
