# Supplementary material for: Molecular mobility on graphene nanoroads
Source: Sci Rep. 2015 Aug 5;5:12848. doi: 10.1038/srep12848 (PMC4525374; doi:10.1038/srep12848)
Supplement: Supplementary Information [file srep12848-s1.pdf]

Supplementary Information for:

Molecular mobility on graphene nanoroads

*Mehdi Jafary-Zadeh and Yong-Wei Zhang**

Institute of High Performance Computing, A*STAR, Singapore 138632

* Corresponding author E-email: zhangyw@ihpc.a-star.edu.sg.

Two supplementary movies are provided as the following:

Sup_Mov_1_Arm.avi – Movie, MD simulations representing the diffusive motion of C_{60} admolecule on the armchair-edged graphene nanoroad (width ~ 20 Å) at 100 K.

Sup_Mov_2_Zig.avi – Movie, MD simulations representing the diffusive motion of C_{60} admolecule on the zigzag-edged graphene nanoroad (width ~ 20 Å) at 100 K.

These supplementary movies illustrate the effect of edge type on the molecular motion: the C_{60} motion along the zigzag edge is faster than its motion along the armchair edge.
